# Supplementary material for: Addressing obstetricians’ awareness of compassion, communication, and self-care when caring for families experiencing stillbirth: Evaluation of a novel educational workshop using applied drama techniques
Source: PLoS One. 2022 Nov 17;17(11):e0277496. doi: 10.1371/journal.pone.0277496 (PMC9671339; doi:10.1371/journal.pone.0277496)
Supplement: S1 Appendix — (DOCX) [file pone.0277496.s001.docx]

**S1 Appendix Workshop evaluation questionnaire**

| 1. **How many years have you worked in Obstetrics?** |
| --- |
| 1. <5 years |
| 1. 5-10 years |
| 1. >10 years |
| 1. **What is your sex?** |
| 1. Male |
| 1. Female |
| 1. I’d rather not say |
| 1. **Have you previously received any formal training on caring for families experiencing stillbirth?** |
| 1. Yes. Please explain: |
| 1. No |
| 1. **Have you previously received any formal training on caring for families dealing with intrapartum death?** |
| 1. Yes. Please explain: |
| 1. No |
| 1. **Have you been directly involved in an intrapartum death or an intrapartum event-related neonatal death?** |
| 1. Yes, once |
| 1. Yes, more than once |
| 1. No |
| 1. **Have you been present at a delivery where the baby died during or shortly after birth?** |
| 1. Yes, once |
| 1. Yes, more than once |
| 1. No |
| 1. **What type of intrapartum death was this? (Tick all that apply)** |
| 1. Expected and unpreventable (e.g. Lethal fetal abnormality) |
| 1. Unexpected and largely unpreventable (e.g. Placental abruption, cord prolapse, shoulder dystocia) |
| 1. Unexpected and potentially preventable (e.g. Hypoxic ischaemic encephalopathy, intrapartum sepsis) |
| 1. **Have you had to diagnose stillbirth?** |
| 1. Yes, once |
| 1. Yes, more than once |
| 1. No |
| 1. **Have you had to manage stillbirth, including postnatal review?** |
| 1. Yes, once |
| 1. Yes, more than once |
| 1. No |
| 1. **What type of stillbirth was this? (Tick all that apply)** |
| 1. Expected and unpreventable |
| 1. Unexpected |

| 1. **Prior to today’s training workshop, how confident were you in following:** | | | | |
| --- | --- | --- | --- | --- |
| 1. Breaking bad news | | | | |
| (1)  Not confident at all | (2)  Quite unconfident | (3)  Neither confident nor unconfident | (4)  Quite confident | (5)  Very confident |
| 1. Communicating clearly with the family when breaking bad news | | | | |
| (1)  Not confident at all | (2)  Quite unconfident | (3)  Neither confident nor unconfident | (4)  Quite confident | (5)  Very confident |
| 1. Communicating empathetically with the family when breaking bad news | | | | |
| (1)  Not confident at all | (2)  Quite unconfident | (3)  Neither confident nor unconfident | (4)  Quite confident | (5)  Very confident |
| 1. Recognising the emotional needs of the family | | | | |
| (1)  Not confident at all | (2)  Quite unconfident | (3)  Neither confident nor unconfident | (4)  Quite confident | (5)  Very confident |
| 1. Recognising your own emotional responses | | | | |
| (1)  Not confident at all | (2)  Quite unconfident | (3)  Neither confident nor unconfident | (4)  Quite confident | (5)  Very confident |
| 1. Supporting your colleagues | | | | |
| (1)  Not confident at all | (2)  Quite unconfident | (3)  Neither confident nor unconfident | (4)  Quite confident | (5)  Very confident |
| 1. **Following today’s training workshop, how confident are you in following:** | | | | |
| 1. Breaking tragic news | | | | |
| (1)  Not confident at all | (2)  Quite unconfident | (3)  Neither confident nor unconfident | (4)  Quite confident | (5)  Very confident |
| 1. Communicating clearly with the family | | | | |
| (1)  Not confident at all | (2)  Quite unconfident | (3)  Neither confident nor unconfident | (4)  Quite confident | (5)  Very confident |
| 1. Communicating empathetically with the family | | | | |
| (1)  Not confident at all | (2)  Quite unconfident | (3)  Neither confident nor unconfident | (4)  Quite confident | (5)  Very confident |
| 1. Recognising the emotional needs of the family | | | | |
| (1)  Not confident at all | (2)  Quite unconfident | (3)  Neither confident nor unconfident | (4)  Quite confident | (5)  Very confident |
| 1. Recognising your own emotional responses | | | | |
| (1)  Not confident at all | (2)  Quite unconfident | (3)  Neither confident nor unconfident | (4)  Quite confident | (5)  Very confident |
| 1. Supporting your colleagues | | | | |
| (1)  Not confident at all | (2)  Quite unconfident | (3)  Neither confident nor unconfident | (4)  Quite confident | (5)  Very confident |
| 1. **To what degree did today’s training workshop cover the following attributes needed when caring for families experiencing stillbirth:** | | | | |
| 1. Self-awareness | | | | |
| (1)  Too little | (2)  Little | (3)  Just right | (4)  A lot | (5)  Too much |
| 1. Authenticity | | | | |
| (1)  Too little | (2)  Little | (3)  Just right | (4)  A lot | (5)  Too much |
| 1. Your own emotional responses | | | | |
| (1)  Too little | (2)  Little | (3)  Just right | (4)  A lot | (5)  Too much |
| 1. The role of verbal communication | | | | |
| (1)  Too little | (2)  Little | (3)  Just right | (4)  A lot | (5)  Too much |
| 1. The role of nonverbal communication | | | | |
| (1)  Too little | (2)  Little | (3)  Just right | (4)  A lot | (5)  Too much |
| 1. Active listening | | | | |
| (1)  Too little | (2)  Little | (3)  Just right | (4)  A lot | (5)  Too much |
| 1. Building of rapport | | | | |
| (1)  Too little | (2)  Little | (3)  Just right | (4)  A lot | (5)  Too much |
| 1. Building of resilience | | | | |
| (1)  Too little | (2)  Little | (3)  Just right | (4)  A lot | (5)  Too much |

| 1. **How would you rate the following aspects of the training workshop:** | | | | |
| --- | --- | --- | --- | --- |
| 1. The mode of delivery (simulation) | | | | |
| (1)  Very poor | (2)  Poor | (3)  Average | (4)  Good | (5)  Very good |
| 1. The length of the training | | | | |
| (1)  Very poor | (2)  Poor | (3)  Average | (4)  Good | (5)  Very good |
| 1. The setting of the training | | | | |
| (1)  Very poor | (2)  Poor | (3)  Average | (4)  Good | (5)  Very good |
| 1. The facilitators | | | | |
| (1)  Very poor | (2)  Poor | (3)  Average | (4)  Good | (5)  Very good |
| 1. The stories used | | | | |
| (1)  Very poor | (2)  Poor | (3)  Average | (4)  Good | (5)  Very good |
| 1. The relatability of the training to practice | | | | |
| (1)  Very poor | (2)  Poor | (3)  Average | (4)  Good | (5)  Very good |
| 1. Ability to actively participate in the training | | | | |
| (1)  Very poor | (2)  Poor | (3)  Average | (4)  Good | (5)  Very good |
| 1. Clarity of learning points | | | | |
| (1)  Very poor | (2)  Poor | (3)  Average | (4)  Good | (5)  Very good |
| 1. **Would you recommend todays’ workshop to your colleague?** | | | | |
| 1. Yes |  |  |  |  |
| 1. No |  |  |  |  |
| 1. **Please provide one positive comment and one point for improvement on the training workshop** | | | | |
| Positive comments: | | | | |
| Points for improvement: | | | | |
| 1. **Do you have any other comments or feedback on the training workshop?** | | | | |
|  | | | | |
